# Regulation of cancer stem cell properties, angiogenesis, and vasculogenic mimicry by miR-450a-5p/SOX2 axis in colorectal cancer

**DOI:** 10.1038/s41419-020-2361-z

**Published:** 2020-03-06

**Authors:** Jiaxuan Chen, Shengyuan Chen, Linghao Zhuo, Yin Zhu, Haoxuan Zheng

**Affiliations:** Guangdong Provincial Key Laboratory of Gastroenterology, Department of Gastroenterology, Nanfang Hospital, Southern Medical University, Guangzhou, 510515 China

**Keywords:** Cancer stem cells, Colorectal cancer, Cell growth, Cell migration

## Abstract

Growing evidence indicates that a small number of cancer cells express stem cell markers and possess stem cell-like properties that promote malignant progression. Sex-determining region Y-box2 (SOX2) is a stem cell transcription factor essential for maintaining the properties of cancer stem cell (CSC). As CSC properties have been associated with angiogenesis and vasculogenic mimicry (VM), we aimed to comprehensively investigate whether SOX2 regulates CSC properties, angiogenesis, and VM in colorectal carcinoma (CRC) and its potential mechanism in this study. For this study, sphere formation assay, flow cytometry, cell survival analysis, tube formation, 3D culture, immunoblot, mouse model, and luciferase reporter assay were performed in vivo and in vitro. Expressions of SOX2 and miR-450a-5p in CRC tissue samples were examined through immunohistochemistry. First, the expression of SOX2 was not only associated with poor differentiation and prognosis but also promoted angiogenesis and VM. Knockdown of SOX2 ceased stemness properties, angiogenesis, and VM, along with decreased expression of CD133, CD31, and VE-cadherin as observed in functional experiments. Downregulation of SOX2 was found to inhibit tumorigenesis in vivo. Second, miR-450a-5p suppressed the expression of SOX2 by targeting its 3’UTR region directly and hence restrained SOX2-induced CSC properties, angiogenesis, and VM. Moreover, SOX2 overexpression preserved the miR-450a-5p-induced inhibition of CRC properties, angiogenesis, and VM. Finally, clinical samples exhibited a negative correlation between miR-450a-5p and SOX2. Patients with higher SOX2 and lower miR-450a-5p expressions had a poorer prognosis than patients with inverse expressions. Conclusively, we elucidated a unique mechanism of miR-450a-5p-SOX2 axis in the regulation of stemness, angiogenesis, and VM, which may act as a potential therapeutic practice in CRC.

## Introduction

Colorectal cancer (CRC) is the third most common type of cancer worldwide and the fourth most common cause of death in China^1^. Growing evidence indicates the occurrence of a small number of cancer stem cells (CSCs) in tumor tissues that might be responsible for the development and recurrence of tumor and appear to be a promising treatment for curing cancer^2^. Recent studies have reported the CSCs to be the most strong angiogenic cells in tumor, and the acquisition of CSC properties is relevant to endothelial vessel formation, indicating a close link between cancer cell stemness and vasculature^3,4^.

Angiogenesis is the first mode of vascularization that was discovered and implicated in cancer cell stemness. In brain tumors, stem-cell-like glioma cells were reported to promote angiogenesis and tumor growth by way of increased vascular endothelial growth factor (VEGF) secretion^5,6^. Vasculogenic mimicry (VM) may also play a key role as an alternative pathway for blood supply when angiogenesis is inhibited^7^. VM is the unique ability of the aggressive tumor cells to form periodic acid-Schiff-positive and CD31-negative cells that line up VM networks in vivo and form tubular structures and patterned networks in 3D cultures in vitro^8^. VM has been reported to occur in numerous types of aggressive tumors including CRC and also to be involved in the acquisition of CSC properties by inducing epithelial-to-mesenchymal transition (EMT)^4^. Dang and Ramos reported tumor cells with CSC attributes to be able to form vascular-like structures in oral squamous cell carcinoma^9^. In fact, angiogenesis and VM coexist commonly within aggressive tumors, both of which are relevant to the acquisition of CSC properties.

Complex networks involving several transcription factors, such as Nanog, Oct4, SOX2, and various miRNAs, have been identified to regulate CSC properties^10,11^. SOX2 performs important functions during embryonic development and is required for maintenance of CSC phenotypes^12,13^, which leads to aggressive tumor growth, invasion, and resistance to conventional therapy in various types of cancers^14,15^. Multiple miRNAs have been reported to be involved in the stemness maintenance. In the case of CRC, factors such as miR-200c, miR-638, and miR-371–5p have been reported to regulate SOX2-induced CSC properties^16–18^. Several recent studies have demonstrated that stemness-associated genes, such as OCT4 and Klf4, are also implicated in modulating angiogenesis and VM^19,20^. Whether stemness-related factors such as SOX2 can modulate stemness properties, angiogenesis, and VM is yet to be explored.

In this study, we investigated if SOX2 modulated CSC properties, angiogenesis, and VM in CRC and further explored its underlying mechanisms in vivo and in vitro. We found that miR-450a-5p-SOX2 axis played an important role in CRC stemness, angiogenesis, and VM, which may act as a potential therapeutic treatment.

## Materials and methods

### Cell culture

Human CRC cell lines SW480 and SW620 were obtained from the American Type Culture Collection (ATCC) and authenticated according to the recommendations of ATCC and were cultured in an RMPI-1640 medium containing 10% fetal bovine serum (Gibco, USA) in 5% CO_2_ at 37 °C. Human embryonal kidney 293 cells and primary human umbilical vein endothelial cells (HUVECs) were preserved at Nanfang Hospital, Southern Medical University (Guangzhou, China) and cultivated in a DMEM medium supplemented with 10% FBS.

### Tissue microarray and CD31/periodic acid-Schiff double-staining

Glass-slide tissue arrays including 90 pairs of CRC tissues were purchased from the Shanghai Outdo Biotech (Shanghai, China), and either immunohistochemistry (IHC) or fluorescence in situ hybridization (FISH) were performed using the tissue microarray slides. The intensity of staining of malignant cells was scored as follows to analyze the levels of protein expression: + (no staining), ++ (weak staining), +++ (moderate staining), and ++++ (strong staining). A score of intensity >++ was classified as high expression, whereas ≤++ was considered as low expression^21^. For a double-staining assay, the slide was stained with CD31 through IHC and then counterstained with periodic acid-Schiff (sigma). The presence of VM refers to the PAS-positive and CD31-negative CRC cells that form tubular structures and patterned networks^22^.

### MicroRNA sequencing

Total RNA from SW480 and SW620 was extracted using the TRIzol reagent (Takara Bio, Japan) and used for microRNA sequencing analysis (Aksomics, China).

### Quantitative real-time PCR, western blot, lentiviral transfection, cell proliferation and invasion assays, and flow cytometry

qRT-PCR, Western blot, lentiviral transfection, cell proliferation and invasion assays, and flow cytometry were all performed as described earlier^23^. qRT-PCR primers for miRNAs, snRNA U6, SOX2, and GAPDH were procured from GeneCopoeia. The antibodies are listed in the Supplementary file.

### Sphere formation assay

Cells were cultured at 80% confluence and then digested using trypsin into a single cell suspension. Thereafter, 10^3^ cells were resuspended in 1 ml serum-free DMEM-F12 medium containing EGF (10 μg/l), basic fibroblast growth factor (20 μg/l), and B27 supplement, and then transferred into each well of a 24-well ultra-low-attachment plate (Corning, USA). Incubation was conducted at 37 °C for 8–10 days, after which spheres were quantified under a light microscope (X100).

### Cell survival analysis

Cells in the serum-free medium were seeded in a 96-well plate (10^4^ cells/well) for about 8–12 h overnight. Then chemotherapeutic agent (SN-38: 4 μM/5-Fu: 15μg/ml/ OXaliplatin: 60 μM) for CRC patients was added into the medium for 48 h. Cell Counting Kit-8 (Dojindo, Japan) was used to analyze cell survival according to the manufacturer’s instructions.

### HUVEC tube-formation assay

Cells transfected with SOX2 expression lentiviruses/lentiviral shRNA or miR-450a-5p mimics/inhibitor were cultured as described hereinbefore. On reaching 80% cell confluence, the culture medium was replaced with serum-free DMEM. Post another 24-h culture, the supernatant was collected as a conditioned medium and stored at −80 °C. Each well of a 96-well plate was precoated with 50 μl of Matrigel (BD, USA) for conducting the tube-formation assay, which was allowed to polymerize for 30 min at 37 °C. HUVECs were suspended at a density of 1.5 × 10^5^ cells/ml in different conditional media, and 100 μl of the cell suspensions was added to each Matrigel-coated well. The formation of capillary-like structures after about 6 h was captured using a light microscope (X100).

### Three-dimensional (3D) culture

VM formation was tested using a 3D culture containing Matrigel (BD, USA) in vitro. Culture plates with 24 wells were coated with Matrigel (100 μl/well). The CRC cells were trypsinized and suspended in the complete medium at 2.5 × 10^5^ cells/ml, transferred onto the surface of Matrigel at 1 ml/well, and incubated at 37 °C for 48 h. The number of tube-like structures was calculated under the light microscope (X100).

### Luciferase reporter assay

Luciferase reporter plasmids were produced through ligation of oligonucleotides containing the wild-type (Wt) or mutant (Mut) putative target site of the SOX2 3’UTR, transfected into the GV272 vectors (Genechem, China). The 293T cells were cotransfected with the luciferase vectors and miR-450a-5p mimics or negative control plasmid using Lipofectamine 2000 (Invitrogen). Post 48 h, 293T cells were lyzed, and luciferase activity was analyzed using the Dual Luciferase Reporter Assay Kit (Promega) as per the manufacturer’s instructions.

### Tumorigenesis in vivo

Four-six-week-old female athymic BALB/c nude mice were obtained from the Central Laboratory of Animal Science at Southern Medical University and housed in laminar flow cabinets under specific pathogen-free conditions. Xenograft tumors were produced by subcutaneous injection of 1 × 10^6^ cells. Tumor volume was assessed by an external caliper and calculated using the equation (L × W^2^)/2 constantly. All experimental procedures using animals were conducted as per the animal protocol approved by the Animal Care and Use Committee of Southern Medical University.

### Statistical analysis

Statistical analysis was conducted using SPSS 21.0. Expressional difference of each molecule in ranked data was analyzed using chi-square test. Difference in IHC scoring was calculated by Mann–Whitney test, while significant changes among different groups were evaluated through one-way ANOVA or independent samples *t*-test. Correlation coefficient was calculated using the Spearman method. Survival curves were plotted on the basis of the follow-up data by Kaplan–Meier method. *P*-values ≤ 0.05 were considered statistically significant.

## Results

### SOX2 expression correlated with angiogenesis and VM in CRC predicting malignant progression and poor prognosis

**Fig. 1:**
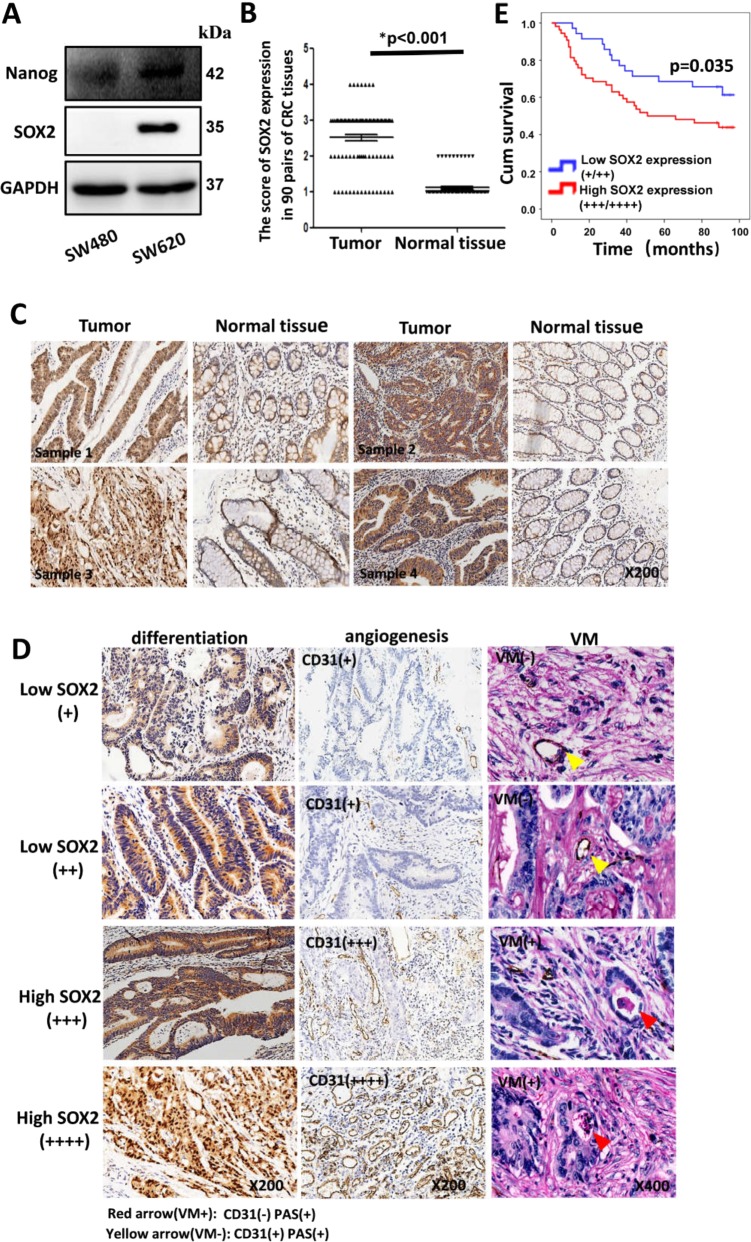
SOX2 expression correlated with tumor differentiation, vascularity, and VM in CRC. The expression of seven ESC markers (Nanog, SOX2, Rex1, KLF4, Bmi1, P63, Oct4) was evaluated in human CRC cell lines through western blot (**a**). CRC tissue arrays were obtained and IHC was performed with SOX2 antibody. The score of SOX2 expression in 90 pairs of CRC tissues was evaluated by two observers (**b**, **c**). IHC was employed to evaluate SOX2 and CD31 expression in CRC specimens. For double-staining assay, the slide was stained with CD31 by IHC and then counterstained with periodic acid-Schiff (**d**). Kaplan–Meier survival analysis of overall survival in 90 CRC patients was plotted (**e**).

Stemness-associated genes such as Nanog, SOX2, and Bmi1 have been reported to regulate CSC properties. Recent studies have demonstrated the acquisition of CSC phenotypes and functions as implicated in angiogenesis and VM formation^3,4^. First, the expressions of seven embryonic stem cells (ESC) markers, namely, Nanog, SOX2, Rex1, Klf4, Bmi1, P63, and Oct4, in human CRC cell lines were evaluated through Western blot. Our findings indicated a higher expression of Nanog and SOX2 in SW620 than SW480 (Fig. 1a), whereas the rest of the markers were observed to be lower in SW620 than SW480 (Supplement Fig. 1A). SOX2 was determined to be a potential factor as its expression was significantly upregulated in the highly metastatic CRC cell line SW620. To compare the ability of stem cell properties and vasculature mimicry formation between SW620 and SW480, sphere formation, cell viability, proliferation, invasion, tube formation assay, and 3D culture were performed. Compared with SW480, SW620 exhibited higher abilities in sphere formation, cell viability, growth, invasion, angiogenesis, and VM formation (Supplement Fig. 1 B–H), demonstrating that endogenous SOX2 promotes the ability of stem cell properties and vasculature mimicry formation.

We evaluated the expression of SOX2 gene using IHC in a CRC tissue array (90 pairs of CRC tissues). SOX2 expression in CRC tissue samples (82.2%, 74 of 90) was found to be significantly higher than that in the corresponding normal tissues (Fig. 1b, c). SOX2 expression was significantly correlated with pathological grade, lymph node metastasis, AJCC stage, tumor size, and VM formation (Supplement Table. 1). Accordingly, overexpression of SOX2 resulted in poor differentiation and the occurrence of angiogenesis and VM (Fig. 1d). The survival duration of patients with higher SOX2 was shorter than patients with lower SOX2 expression (Fig. 1e). Overall, these results indicate that the higher expression of SOX2 is related to progression of malignancy and poor prognosis among CRC patients.

### Knockdown of SOX2 restrained CSC properties, angiogenesis, and VM in vitro and in vivo

**Fig. 2:**
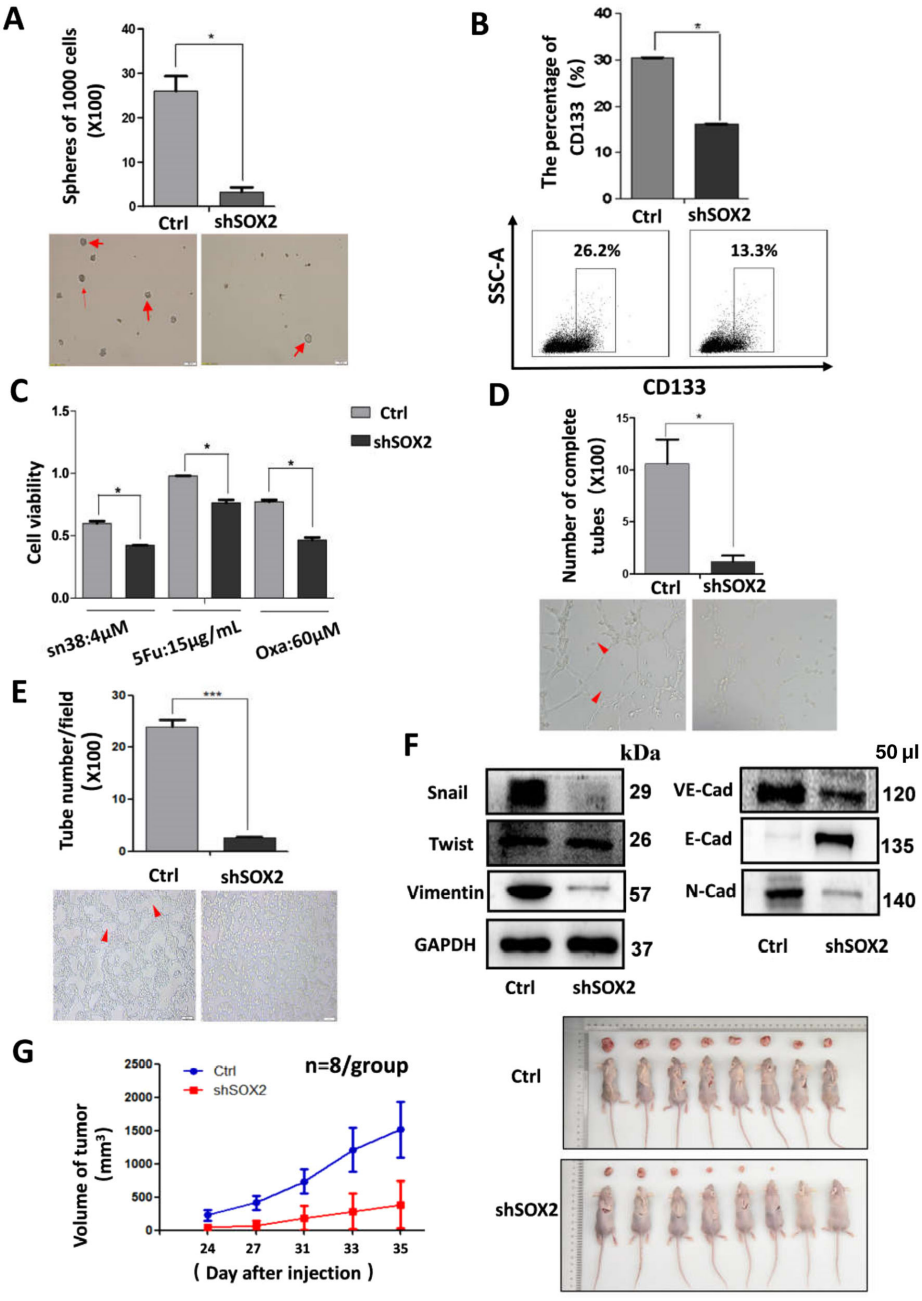
Knockdown of SOX2 expression in SW620 restrained CRC stemness, angiogenesis, VM, and tumor growth. SW620 cells stably expressing SOX2 shRNA or control shRNA construct were digested into single cell suspension. Then sphere formation assay was performed as indicated (**a**). 4 × 10^5^ SW620 cells were incubated with CD133-APC for 30 min at 4 °C, and then analyzed by flow cytometry (**b**). Transfected SW620 cells were seeded in 96-well plate (10,000 cells/well), and treated with different drug as indicated for 48 h, and cell viability was analyzed (**c**). HUVECs were suspended at a density of 1.5 × 10^5^ cells/ml in the different supernatants (derived from cultured medium with SOX2-silenced cells or control cells), and 100 μl of the cell suspensions were added to each Matrigel-coated well. After 6 h, the formation of capillary-like structures was captured under a light microscope (**d**, x100). SW620 cells (2.5 × 10^5^/well) were plated onto the surface of Matrigel and incubated at 37 °C for 48 h. The numbers of tube-like structures were measured a light microscope (**e**, x100). Western blot was performed to determine expression of EMT markers and VM-related factor (**f**). All of them were corresponding to the same blot as the GAPDH loading control. Tumor volume was periodically measured for each mouse and growth curves were plotted (**g**). All data are represented as mean ± SD compared to control cells or group. Experiments were performed in triplicate. *P < 0.05.

SOX2 plays an important role in embryonic development and is required for the maintenance of CSC phenotypes^12,13^, which may also regulate angiogenesis and VM^19,20^. Cells transfected with SOX2 expression lentiviruses or lentirivial shRNA were used for sphere formation assay, cell survival analysis, tube formation assay, and 3D culture to analyze whether SOX2 directly regulated CRC stemness, angiogenesis, and VM (Supplement Fig. 2A). Knockdown of SOX2 was found to reduce the number of sphere formations and the expression of CRC stem cell markers CD133 (Fig. 2a, b). Cell viability was also reduced in cells transfected with SOX2 shRNA as compared to their control cells when treated with SN38, 5-Fu or Oxaliplatin (Oxa) (Fig. 2c). Tube formation assay showed that the downregulation of SOX2 inhibited the potential of angiogenesis (Fig. 2d). Furthermore, 3D culture assay showed that downregulation of SOX2 suppressed VM formation with reduced expression of VM-associated marker VE-cadherin (Fig. 2e, f). Cessation of SOX2 expression was observed to inhibit the induction of EMT and the abilities of cell proliferation and invasion (Fig. 2f and Supplement Fig. 4B), whereas its overexpression appeared to have the opposite effect (Supplement Fig. 3A–F and Supplement Fig. 4A).

To further validate the above findings in vivo, the SOX2-silenced and control cells were inoculated into nude mice. Tumor volumes and growth rates were found to have significantly decreased in tumors generated from SOX2-silenced cells (Fig. 2g). Overall, these findings proved that SOX2 played an essential part in the acquisition of CRC stemness and the occurrence of angiogenesis and VM.

### miR-450a-5p acts as a novel upstream regulator for SOX2 by directly targeting its 3′UTR

**Fig. 3:**
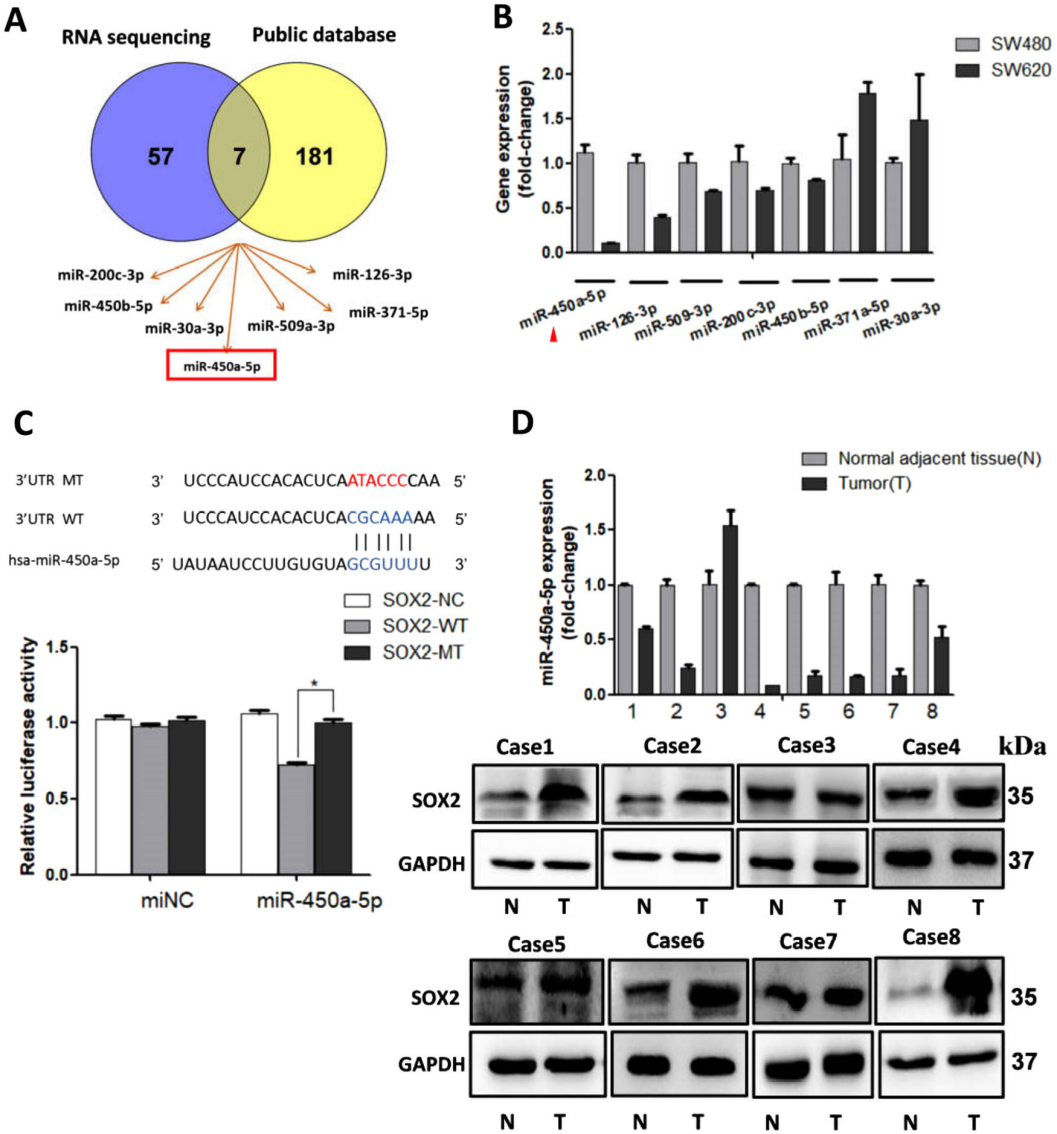
miR-450a-5p inhibited SOX2 expression by directly targeting its 3′UTR. The data from RNA sequencing and public database (microRNA, TargetScan and miRWalk) were overlapped using Venny tool (**a**). qRT-PCR was utilized to further detected the expression of possible miRNAs in CRC cells (**b**). miR-450a-5p and its putative binding sequences in the 3′UTR of SOX2. A mutation was generated in the complementary site that binds to the seed region of miR-450a-5p. A wild/mutant type SOX2 3′UTR reporter (or control construct) and miR-450a-5p plasmid (or control plasmid) were transduced into 293T cells, and luciferase activity was assessed (**c**). qRT-PCR and western blot were used to determine miR-450a-5p and SOX2 expression in 8 paired human colorectal cancer, respectively (**d**). All data are represented as fold-change±SD compared to control cells or group. Experiments were performed in triplicate. *P < 0.05.

Several studies have reported that miRNAs play significant roles in cell differentiation, tumor vascularity, and tumorigenicity by way of posttranscriptional regulation^16,24^. In this study, we aimed to investigate a potential miRNA that directly binds to SOX2. MiRNA sequencing analysis showed that 64 miRNAs were downregulated in SW620 compared with SW480 (Supplement Table. 2). Publicly available algorithms (microRNA, TargetScan, and miRWalk) were additionally used to show that about 188 miRNAs are potential upstream mediators of SOX2. Later, miR-450a-5p, miR-126–3p, miR-509–3p, miR-200c-3p, miR-450b-5p, miR-371–5p, and miR-30a-3p were selected using Venny tool (Fig. 3a). qRT-PCR was utilized to further identify the expressions of these seven miRNAs in CRC cells. Of the studied miRNAs, miR-450a-5p was found to be downregulated in SW620 to a larger extent than the rest of them (Fig. 3b). Bioinformatic analysis conducted using TargetScan indicated that 3′UTR of SOX2 comprises predicted binding sites for miR-450a-5p (Fig. 3c). Then, a dual-luciferase reporter assay was conducted to further study whether miR-450a-5p directly targets SOX2. Ectopic overexpression of miR-450a-5p was found to inhibit wild-type SOX2 3’UTR luciferase activity, but did not inhibit the mutant luciferase activity (Fig. 3c). Consistent with these results, miR-450a-5p level was observed to be inversely correlated with SOX2 expression in eight CRC patients (Fig. 3d). Overall, miR-450a-5p is reported to negatively regulate SOX2 by directly binding to its 3′UTR sequences.

### miR-450a-5p also modulated CRC stemness, angiogenesis, and VM

**Fig. 4:**
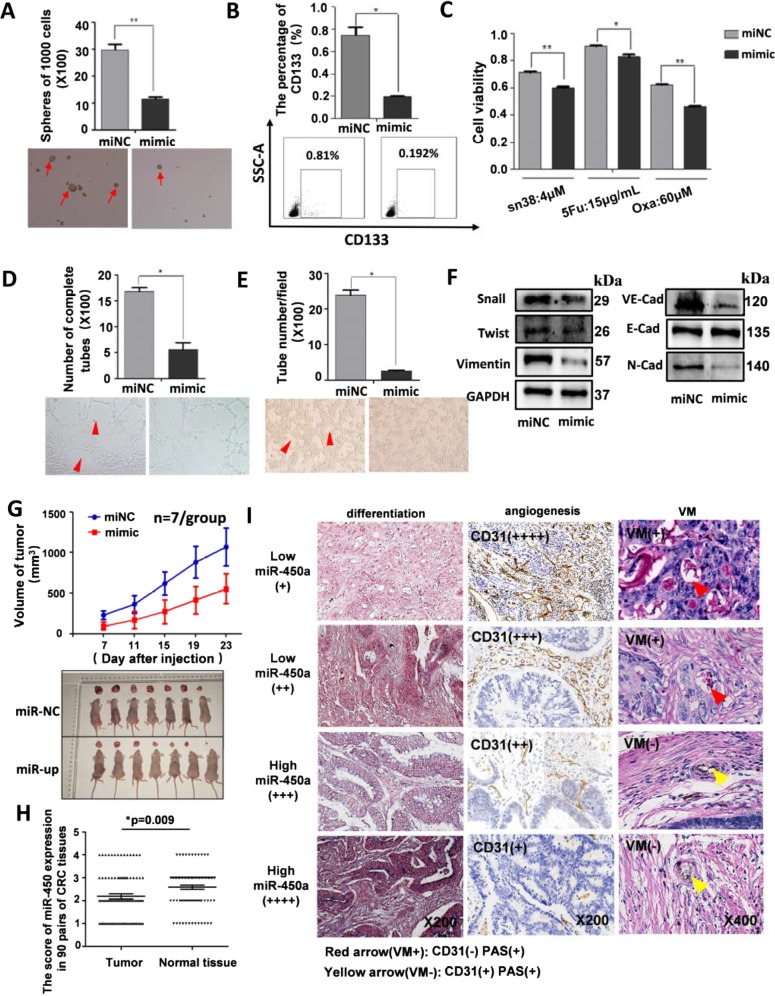
Overexpression of miR-450a-5p suppressed CSC properties, vasculature, and tumor growth. SW620 cells stably expressing miR-450a-5p or control construct were digested into single cell suspension. Then sphere formation assay was performed as indicated (**a**). 4 × 10^5^ SW620 cells were incubated with CD133-APC for 30 min at 4 °C and analyzed by flow cytometry (**b**). Cells were seeded in 96-well plate (10,000 cells/well), and then treated with different drug as indicated for 48 h, and cell viability was analyzed (**c**). HUVECs were suspended at a density of 1.5 × 10^5^ cells/ml in the different supernatants (derived from cultured medium with miR-450a-5p-overexpressed cells or control cells), and 100 μl of the cell suspensions were added to each Matrigel-coated well for 6 h, and the formation of capillary-like structures was captured under a light microscope (**d**, x100). SW620 cells (2.5 × 10^5^/well) were plated onto the surface of Matrigel and incubated at 37 °C for 48 h. The numbers of tube-like structures were measured under a light microscope (**e**, x100). Western blot was performed to determine expression of EMT markers and VM-related factor (**f**). All of them were corresponding to the same blot as the GAPDH loading control. Tumor volume was periodically measured for each mouse and growth curves were plotted (**g**). miR-450a-5p expression in 90 pairs of CRC tissues was evaluated by FISH and scored by two observers (**h**). CRC tissue arrays were obtained and IHC was performed (**i**). All data are represented as mean ± SD compared to control cells or group. Experiments were performed in triplicate. *P < 0.05, **P < 0.01.

As miR-450a-5p inhibited SOX2 expression, which is involved in CRC stemness and vascularity, we hypothesized that it might operate as a suppressor. We also found that increased expression of miR-450a-5p significantly ceased CRC stemness including the sphere-forming capacity, CD133 expression (Fig. 4a, b), chemoresistance (Fig. 4c), and cell proliferation and invasion (Supplement Fig. 6B). Upregulation of miR-450a-5p was found to decrease the formation of endothelial vessels and VM (Fig. 4d, e). Lower expression of mesenchymal markers (Vimentin, Snail, and N-cadherin) and VE-cadherin was additionally observed in cells transfected with miR-450a-5p mimic (Fig. 4f). However, silenced miR-450a-5p expression was observed to contribute to stemness properties, angiogenesis, and VM (Supplement Fig. 5A–F). In addition, mice inoculated with cells overexpressing miR-450a-5p were found to have smaller tumor burdens and slower tumor growth (Fig. 4g).

Furthermore, tissue array indicated that miR-450a-5p expression decreased in 42 of 90 CRC specimens measured by FISH, while it increased in 19 of 90 CRC specimens (Fig. 4h). A significant difference was observed between miR-450a-5p expression level and histological grade, lymph metastasis, clinical stage, or VM (Supplement Table. 3). Patients exhibiting lower expression of miR-450a-5p showed a higher potential for poor differentiation, angiogenesis, and VM formation observed in clinic (Fig. 4i). To summarize, upregulation of miR-450a-5p restrained CRC stemness, angiogenesis, and VM in vivo and in vitro.

### SOX2 overexpression rescued miR-450a-5p-induced inhibition of CRC properties and vasculature

**Fig. 5:**
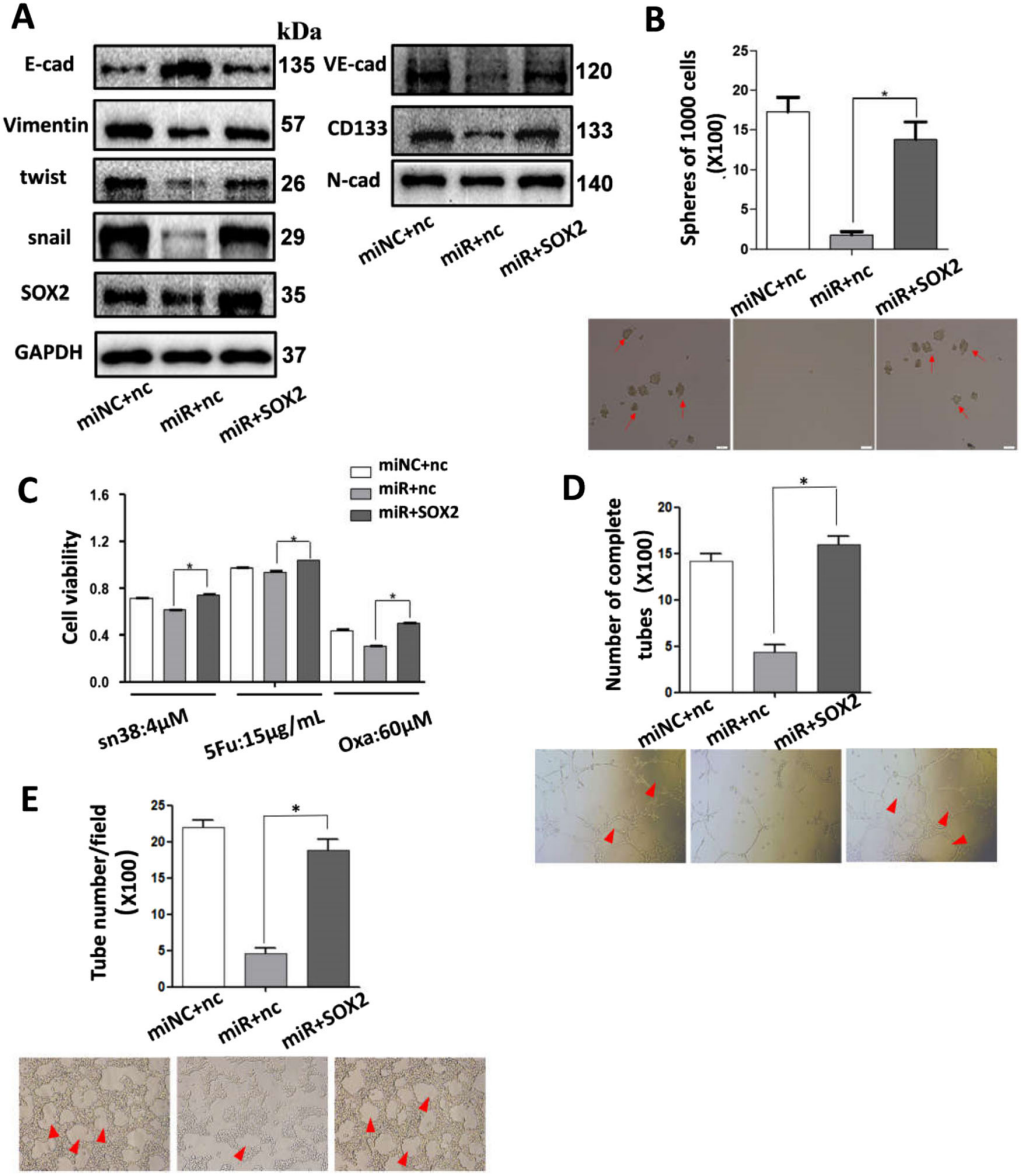
SOX2 overexpression rescued the miR-450a-5p-induced inhibition of CRC stemness, angiogenesis, and VM in SW620. Western blot was performed to determine expression of EMT markers, VE-cadherin and CD133 (**a**). SW620 cells stably cotransfected with miR-450a-5p and SOX2 expression lentiviruses were digested into single cell suspension. Then sphere formation assay was performed as indicated (**b**). Cells were treated with different drug as indicated for 48 h, and cell viability was analyzed (**c**). Different cell supernatant with HUVECs were added to each Matrigel-coated well for 6 h, and the formation of capillary-like structures was captured under a light microscope (**d**, ×100). Cells were plated onto the surface of Matrigel and incubated at 37 °C for 48 h and then the numbers of tube-like structures were measured under a light microscope (**e**, x100). All data are represented as mean ± SD compared to control cells or group. Experiments were performed in triplicate. *P < 0.05. miRNC:miR-450a-5p lentiviruses negative control; miR: miR-450a-5p expression lentiviruses; nc: SOX2 lentiviruses negative control; SOX2: SOX2 expression lentiviruses.

To further confirm whether miR-450a-5p mediated its CSC properties and vascularity using SOX2, miR-450a-5p and SOX2 expression lentiviruses was cotransfected into SW620; the expression of SOX2 was detected by WB (Fig. 5a). Upregulation of SOX2 was found to partially abrogate overexpressed-miR-450a-5p-induced inhibition of spheres formation and cell viability (Fig. 5b–c). Most importantly, higher amounts of angiogenesis and VM formation were observed in SOX2-overexpressing cells compared with control cells (Fig. 5d–e). Besides, reduced CD133 expression, VE-cadherin, and mesenchymal markers in cells transfected with miR-450a-5p lentiviruses could be reversed by SOX2 expression (Fig. 5a). These findings proved that miR-450a-5p induced the inhibition of CRC stemness, angiogenesis, and VM, partially at least, by directly downregulating SOX2 expression.

### MiR-450a-5p negatively correlated with SOX2 in vivo, whereas SOX2 positively correlated with CD133, CD31, and VE-cadherin

**Fig. 6:**
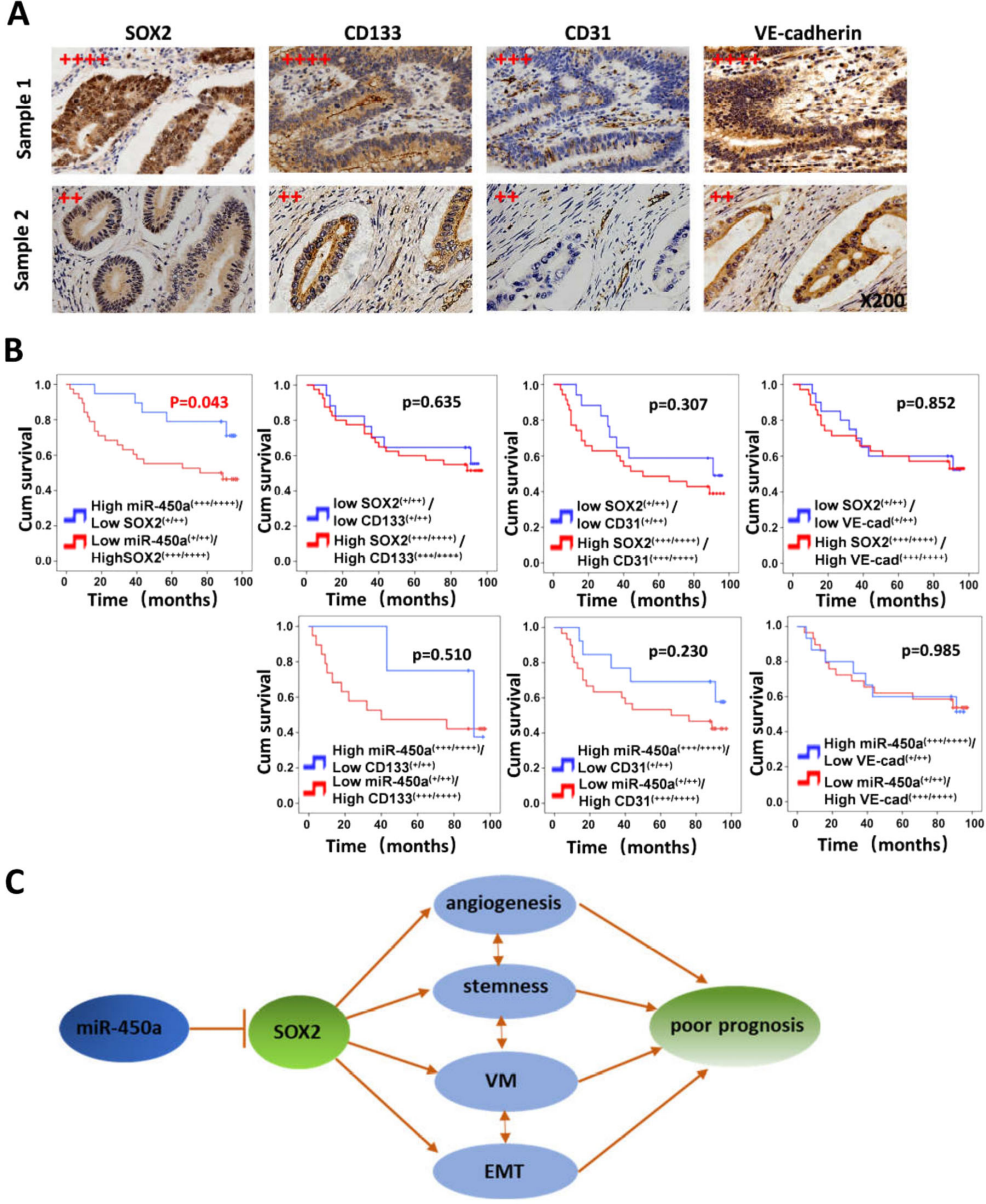
MiR-450a-5p negatively correlated with SOX2, while SOX2 expression was positively associated with the expression of CD133, CD31, and VE-cadherin. CRC tissue arrays were obtained and IHC was performed (**a**). Sample 1 and Sample 2 were represented for CRC specimens with high SOX2 expression and low SOX2 expression, respectively. Kaplan–Meier survival analysis of overall survival of 90 CRC patients was plotted (**b**). Log-rank test was used to calculate *P*-values. MiR-450a-5p-SOX2 axis regulates CSC properties, angiogenesis and VM (**c**).

In this study, miR-450a-5p was found to repress SOX2-induced CRC stemness and vasculature, accompanied by reduced expressions of CD133, CD31, and VE-cadherin. To further study their correlations in vivo, FISH and IHC were performed in tissue array. As was expected, an inverse association between miR-450a-5p and SOX2 was observed (Table 1), whereas SOX2 was found to be positively correlated with CD133, CD31, and VE-cadherin (Table 2, Fig. 6a). The Kaplan–Meier survival analysis indicated that patients with higher SOX2 (+++/++++) and lower miR-450a-5p (+/++) expressions were subjected to poorer prognosis compared with patients exhibiting inverse expression (Fig. 6b). Nevertheless, no significant difference was observed in prognosis between patients with lower as well as higher expressions of SOX2 and CD133/VE-cadherin/CD31. Overall, these findings implied the expressions of miR-450a-5p and SOX2 correlated in clinical samples and may predict prognosis in CRC patients (Fig. 6c).

**Table 1:** Correlation between SOX2 and miR-450a.

		SOX2 scoring		Correlation coefficient (*R*)
		**Low expression (**+**++/++++) (*n*** = **49)**	**High expression (**+/**++) (*n*** = **29)**	
miR-450a Scoring	Low expression (+/++) (*n* = 48)	10	38	*R* = −0.43
	High expression (+/++) (*n* = 30)	19	11	*P* < 0.001

**Table 2:** Correlation between SOX2, CD133, CD31 and VE-cad.

		SOX2 scoring		Correlation coefficient (*R*)
		**Low expression (+/++) (*n*** = 29)	**High expression (+++/++++) (*n*** = 49)	
CD133 Scoring	Low expression (+/++) (*n* = 26)	17	9	*R* = 0.415
	High expression (+++/++++) (*n* = 52)	12	40	*P* = 0.000
CD31 Scoring	Low expression (+/++) (*n* = 31)	17	14	*R* = 0.315
	High expression (+++/++++) (*n* = 46)	11	35	*P* = 0.005
VE-cad Scoring	Low expression (+/++) (*n* = 34)	20	14	*R* = 0.394
	High expression (+++/++++) (*n* = 44)	9	35	*P* = 0.000

## Discussion

Plenty of evidence strongly indicate that human cancer can be categorized as a stem cell disease. As “cancer stem cell” theory postulates, tumor growth is triggered by a small number of cancer stem cells, which represent a minority in a total tumor cell population but can proliferate uncontrollably and differentiate into numerous cell phenotypes^25^. Transcription factors such as SOX2, Nanog, Oct4, and Bmi1 have been recognized to regulate stem cell pluripotency and also contribute to the maintenance of CSCs^10,26,27^. Recent studies have demonstrated the acquisition of CSC phenotypes and functions as implicated in the vasculature^3,4^. Whether stemness-related SOX2 regulates cancer stemness and vasculature needs to be explored further. In this study, miR-450a-5p-SOX2 axis was found to play an important role in the regulation of CRC stemness and vasculature in vivo and in vitro.

SOX2 has been reported to act as an oncogene during cancer progression and is frequently overexpressed in poorly differentiated CRC^28^. Consistently, our study found that high expression levels of SOX2 were significantly associated with poor differentiation and lymph metastasis as well as a more advanced stage of cancer. However, other studies reported SOX2 to be downregulated in gastric cancer, and lower SOX2 expression levels contributed to poorer prognosis^29^. The inconsistent expression phenotype of SOX2 in tumors may be due to carcinoma heterogeneity. Furthermore, SOX2 is responsible for maintaining the CSC properties, resulting in aggressive tumor growth, invasion, and resistance to conventional therapy in various cancers^14,15^. Basu-Roy et al. proved that downregulating SOX2 restrained tumorigenesis in osteosarcoma, and cells with lowered SOX2 levels were not able to form osteospheres and differentiate into mature osteoblasts any further^14^. Ovarian cancer cell lines that failed to express SOX2 were sensitive to carboplatin, cisplatin, and paclitaxel developed resistance following the ectopic expression of SOX2^15^. Similarly, we proved in this study that cessation of SOX2 expression significantly suppressed the sphere-forming capacity, drug resistance, and cell proliferation and invasion, as well as tumorigenesis in vivo. SOX2 indeed plays a crucial role in stemness and malignant progression in CRC.

Mounting evidence suggests that maintenance of CSC properties is essential for angiogenesis and VM^3,4^. Liu et al. found that overexpression of stemness-related OCT4 promoted CSC properties and VM formation, thereby facilitating tumor cell migration and metastasis into the blood vessels, followed by promoting aggressiveness in breast cancer^19^. Evidence reveals that the transcription factor KLF4 not only promotes cancer stem cell-like characteristics in osteosarcoma^30^ but also mediated sprouting angiogenesis via Notch^20^. In retinoblastoma tumor, stemness-related SOX2 was associated with eyes with higher VEGF-A expression and tumor invasiveness^31^. Whether SOX2 is capable of modulating angiogenesis and VM in CRC needs to be explored further. CSCs resulted in a strong angiogenic response by producing much higher levels of VEGF^32^. Our findings indicate that SOX2 promoted the potential of angiogenesis with higher VEGF expression (data not provided) and resulted in the improvement of CD31 levels, which was known to be expressed in highly aggressive tumors^33^. Tumors may develop vascularization through angiogenesis as well as develop alternative pathways such as VM to provide nutritional supply^34^. VM is implicated in CSCs through the induction of EMT, and SOX2 can induce EMT, implying that SOX2 may mediate both CSCs properties and VM^8,28^. VE-cadherin is a hallmark of VM^8^. Interestingly, our findings demonstrated that CRC cells overexpressed SOX2, expressed higher VE-cadherin to induce extracellular matrix remodeling, promoting the formation of VM. Collectively, SOX2 can also induce the occurrence of angiogenesis and VM, implying its diverse function in cancer progression.

Multiple miRNAs have been reported to be involved in stemness maintenance and vascularity formation^35–37^. MiR-200c, miR-638, and miR-371–5p have been reported to regulate SOX2 in CRC^16–18^. In this study, miRNA sequencing was executed to explore a novel miRNA. Then, miR-450a-5p was established as a novel upstream regulator for SOX2 in CRC. Cessation of miR-450a-5p expression could promote tumor growth in liver cancer^38^. However, the function of miR-450a-5p in CRC remains unexplored till date. Overexpression of miR-450a-5p suppressed CSC properties, angiogenesis, VM, and tumorigenesis in vivo and in vitro. Moreover, SOX2 overexpression rescued the inhibition of these effects induced by miR-450a-5p. To further investigate their correlations in vivo, FISH and IHC were used in tissue array. MiR-450a-5p was observed to be negatively correlated with SOX2, whereas SOX2 was found to positively correlated with CD133, CD31, and VE-cadherin. Patients with higher SOX2 (+++/++++) and lower miR-450a-5p (+/++) expression had a poorer prognosis than patients with inverse expression. Conclusively, miR-450a-5p induced the inhibition of CRC stemness, angiogenesis, and VM by directly downregulating SOX2 expression.

On the basis of our findings, it is concluded that miR-450a-5p-SOX2 axis regulates stemness, angiogenesis, and VM in CRC and in vitro. Furthermore, our study provides novel insights into the molecular mechanisms of cancer stemness and vasculature and may be helpful in guiding the strategies targeting miR-450a-5p-SOX2 axis for cancer treatment.

## Supplementary information

Supplementary information

Table S1

Table S2

Table S3

figureS1

figureS2

figureS3

figureS4

figureS5

figureS6

checklist

author contribution form-page1

author contribution form-page2
