# Supplementary material for: Regulation of cancer stem cell properties, angiogenesis, and vasculogenic mimicry by miR-450a-5p/SOX2 axis in colorectal cancer
Source: Cell Death Dis. 2020 Mar 6;11(3):173. doi: 10.1038/s41419-020-2361-z (PMC7060320; doi:10.1038/s41419-020-2361-z)
Supplement: Supplementary file 1 — Supplementary information [file 41419_2020_2361_MOESM1_ESM.docx]

**Supplementary information**

**Antibodies, reagents and transfectants**

Antibodies used in immunoblot assay: anti-GAPDH (#3683, Cell Signaling Technology), anti-SOX2 (#3579, Cell Signaling Technology), anti-Nanog (#4903, Cell Signaling Technology), anti-Bmi1 (#6964, Cell Signaling Technology), anti-Rex1 (ab175429, Abcam), anti-Oct4 (ab181557, Abcam), anti-p63 (ab124762, Abcam), anti-KLF4 (ab72543, Abcam), anti-E-Cadherin (#3195, Cell Signaling Technology), anti-N-Cadherin (#13116, Cell Signaling Technology), anti-Snail (#3879, Cell Signaling Technology), anti-Vimentin (ab92547, Abcam), anti-Twist (ab50581, Abcam), anti-CD31 (#3528, Cell Signaling Technology),and anti-VE-Cadherin (NB110-60978, novusbio).

Antibodies used in immunohistochemistry assay: anti-SOX2 (A0561, ABclonal), anti-CD31 (#3528, Cell Signaling Technology), and anti-VE-Cadherin (NB110-60978, novusbio).

Reagents: 5-Fu (101722, MP Biomedicals), Oxaliplatin (O9512, Sigma-Aldrich), SN-38 (H0165, Sigma-Aldrich), and anti-human CD133 APC (17-1338, Affymetrix).

Transfectants: SOX2 overexpressing Lentivirus and negative control (GOSL0108194, SHANGHAI GENECHEM CO.), SOX2 shRNA lentivirus and negative control (GIEL0108193, SHANGHAI GENECHEM CO.), hsa-miR-450a-1 overexpressing Lentivirus and negative control (GMUL0125483, SHANGHAI GENECHEM CO.), hsa-miR-450a-5p microRNA down lentivirus and negative control (GMDL0125484, SHANGHAI GENECHEM CO.), hsa-miR-450a-5p overexpressing plasmid and negative control (GMUE0125479, SHANGHAI GENECHEM CO.), SOX2 3’UTR-WT plasmid and negative control (GOSE0125480, SHANGHAI GENECHEM CO.), SOX2 3’UTR-MT plasmid and negative control (GOSE0125481, SHANGHAI GENECHEM CO.), SOX2 overexpressing plasmid and negative control (GOSE0177806, SHANGHAI GENECHEM CO.), micrONTM hsa-miR-450a-5p mimic (miR10001545, GUANGZHOU RIBOBIO CO.), micrONTM mimic Negative Control #22 (miR1101, GUANGZHOU RIBOBIO CO.), microOFFTM hsa-miR-450a-5p inhibitor (miR20001545, GUANGZHOU RIBOBIO CO.), and microOFFTM inhibitor Negative Control #22 (miR02101, GUANGZHOU RIBOBIO CO.)

Figure legends

Figure S1. CSC properties and vasculature were evaluated in SW620 and SW480. Stemness-related factors were detected by western blot(A). Spheres formation assay was used to measure the CRC stemness(B). SW480 and SW620 were treated with different chemotherapeutics (SN38,5-Fu and Oxaliplatin) for 48h and then cell viability was analyzed(C). Angiogenesis and VM were performed as indicated(D-E). Western blot was performed to determine expression of epithelial and mesenchymal markers(F). Cell proliferation(G) and invasion (H) were determined by CCK-8 and transwell chamber, respectively. Data are represented as mean±SD compared to control group (SW480). Experiments were performed in triplicate. *P < 0.05.

Figure S2. SOX2 was stably upregulated in SW480 and stably downregulated in SW620. The efficacy of gene transfection was tested by qRT-PCR and western blot. Data are represented as fold-change± SD compared to control cells. Experiments were performed in triplicate. *P < 0.05.

Figure S3. Overexpression of SOX2 in SW480 induced CRC stemness, angiogenesis and VM. SOX2-overexpressed cells and control cells were digested into single cell suspension. Then sphere formation assay was performed as indicated(A). 4x10^5^ cells were incubated with CD133-APC for 30 min at 4℃, and analyzed by Flow Cytometry(B). Transfected cells were treated with different drug as indicated for 48 hours, and cell viability was analyzed (C). HUVECs were suspended at a density of 1.5 × 10^5^ cells/ml in the different supernatants (derived from cultured medium with SOX2-overexpressed cells or control cells), and 100ul of the cell suspensions were added to each Matrigel-coated well. After 6 h, the formation of capillary-like structures was captured under a light microscope (D, x100).2.5x10^5^ cells were plated onto the surface of Matrigel and incubated at 37°C for 48 h. The numbers of tube-like structures were measured a light microscope (E, x100). Western blot was performed to determine expression of EMT markers and VE-cadherin(F). All of them were corresponding to the same blot as the GAPDH loading control. All Data are represented as mean ± SD compared to control cells or group. Experiments were performed in triplicate. *P < 0.05.

Figure S4. SOX2 expression promoted cell proliferation and invasion. SW480(A) and SW620(B) stably transfected SOX2 expression lentiviruses or lentiviral shRNA respectively were digested into single cell suspension. Cells were seeded in 96-well plates containing complete medium and 10ul of CCK8 solution was added to each well. cells (5x10^4^/well) were added to the top chamber in serum-free medium and the bottom chamber was filled with medium containing 10% FBS. Then cells were cultured for 48h and stained with 0.1% crystal violet staining solution. Experiments were performed in triplicate. *P < 0.05.

Figure S5. Loss of miR-450a-5p promoted CRC stemness, angiogenesis and VM. SW480 cells transfected with miR-450a-5p inhibitor or control construct were digested into single cell suspension. Then sphere formation assay was performed as indicated (A). 4x10^5^ cells were incubated with CD133-APC and analyzed by Flow Cytometry(B). Cells were treated with different drug as indicated for 48 hours, and cell viability was analyzed (C). HUVECs were suspended at a density of 1.5 × 10^5^ cells/ml in the different supernatants (derived from culture medium with cells transfected miR-450a-5p inhibitor or control construct), and 100ul of the cell suspensions were added to each Matrigel-coated well. After 6 h, the formation of capillary-like structures was captured under a light microscope (D, x100).2.5x10^5^ cells were plated onto the surface of Matrigel and incubated at 37°C for 48 h. The numbers of tube-like structures were measured a light microscope (E, x100). Western blot was performed to determine expression of EMT markers and VE-cadherin(F). All of them were corresponding to the same blot as the GAPDH loading control. All Data are represented as mean ± SD compared to control cells or group. Experiments were performed in triplicate. *P < 0.05, **P < 0.01.

Figure S6. miR-450a-5p suppressed cell proliferation and invasion. SW480(A) and SW620(B) transfected with miR-450a-5p inhibitor or mimic respectively were digested into single cell suspension. Cells seeded in 96-well plates containing complete medium and 10ul of CCK8 solution was added to each well. cells (5x10^4^/well) were added to the top chamber in serum-free medium and the bottom chamber was filled with medium containing 10% FBS. Then cells were cultured for 48h and stained with 0.1% crystal violet staining solution. Experiments were performed in triplicate. *P < 0.05.
