# Supplementary material for: Regulation of cancer stem cell properties, angiogenesis, and vasculogenic mimicry by miR-450a-5p/SOX2 axis in colorectal cancer
Source: Cell Death Dis. 2020 Mar 6;11(3):173. doi: 10.1038/s41419-020-2361-z (PMC7060320; doi:10.1038/s41419-020-2361-z)
Supplement: Supplementary file 2 — Table S1 [file 41419_2020_2361_MOESM2_ESM.docx]

**Table S1 Correlation between the clinicopathologic characteristics and**

**expression of SOX2**

| **Variables** | | **SOX2 expression** | | ***P-value*** |
| --- | --- | --- | --- | --- |
|  |  | **Score**≥3  **High(n=54)** | **Score**<3  **Low(n=36)** |  |
| **Age(years)** |  |  |  |  |
| < 65 |  | 29 | 18 | 0.730 |
| ≥ 65 |  | 25 | 18 |  |
| **Gender** |  |  |  |  |
| Male |  | 28 | 17 | 0.667 |
| Female |  | 26 | 19 |  |
| **Pathological grade** |  |  |  |  |
| Well-Moderate |  | 42 | 34 | **0.033** |
| Poor |  | 12 | 2 |  |
| **Lymph metastasis** |  |  |  |  |
| no |  | 28 | 27 | **0.027** |
| yes |  | 26 | 9 |  |
| **Distant metastasis** |  |  |  |  |
| no |  | 53 | 36 | 0.965 |
| yes |  | 1 | 0 |  |
| **AJCC stage** |  |  |  |  |
| I/II |  | 28 | 27 | **0.027** |
| III/IV |  | 26 | 9 |  |
| **Tumor size** |  |  |  |  |
| < 5 cm |  | 16 | 19 | **0.027** |
| ≥ 5 cm |  | 38 | 17 |  |
| **Tumor number** |  |  |  |  |
| Single |  | 50 | 33 | 0.872 |
| Multiple |  | 4 | 3 |  |
|  |  |  |  |  |
