# Supplementary material for: Regulation of cancer stem cell properties, angiogenesis, and vasculogenic mimicry by miR-450a-5p/SOX2 axis in colorectal cancer
Source: Cell Death Dis. 2020 Mar 6;11(3):173. doi: 10.1038/s41419-020-2361-z (PMC7060320; doi:10.1038/s41419-020-2361-z)
Supplement: Supplementary file 3 — Table S2 [file 41419_2020_2361_MOESM3_ESM.docx]

| **Name** | **Fold-change （Downregulation）** | **Name** | **Fold-change （Downregulation）** |
| --- | --- | --- | --- |
| **hsa-miR-novel-chr4_53015** | **120.2935** | **hsa-miR-10a-5p** | **3.5357** |
| **hsa-miR-509-3-5p** | **84.7027** | **hsa-miR-34c-5p** | **3.5000** |
| **hsa-miR-514a-3p** | **56.0004** | **hsa-miR-146a-5p** | **3.2387** |
| **hsa-miR-novel-chr3_48581** | **37.1002** | **hsa-miR-378i** | **3.2241** |
| **hsa-miR-1-3p** | **17.9411** | **hsa-miR-542-3p** | **3.1528** |
| **hsa-miR-novel-chr18_24880** | **17.6470** | **hsa-miR-450b-5p** | **3.0661** |
| **hsa-miR-143-3p** | **14.9167** | **hsa-miR-novel-chr9_71425** | **3.0559** |
| **hsa-miR-372-3p** | **14.7000** | **hsa-miR-22-5p** | **3.0455** |
| **hsa-miR-novel-chr10_925** | **13.7857** | **hsa-miR-584-5p** | **3.0256** |
| **hsa-miR-novel-chr4_53489** | **10.9231** | **hsa-miR-148a-3p** | **2.9668** |
| **hsa-miR-30a-5p** | **10.7174** | **hsa-miR-novel-chr2_42316** | **2.9091** |
| **hsa-miR-novel-chr18_24754** | **10.7000** | **hsa-miR-novel-chr12_9069** | **2.9091** |
| **hsa-miR-504-5p** | **8.5000** | **hsa-miR-371a-5p** | **2.9000** |
| **hsa-miR-novel-chr2_44917** | **8.4000** | **hsa-miR-29b-3p** | **2.8261** |
| **hsa-miR-508-3p** | **6.2000** | **hsa-miR-novel-chrX_77307** | **2.8182** |
| **hsa-miR-126-3p** | **5.8623** | **hsa-miR-424-3p** | **2.8144** |
| **hsa-miR-451a** | **5.4211** | **hsa-miR-novel-chr10_376** | **2.7273** |
| **hsa-miR-novel-chr7_64181** | **5.3793** | **hsa-miR-novel-chr2_44988** | **2.6667** |
| **hsa-miR-99a-5p** | **5.3469** | **hsa-miR-203b-3p** | **2.5625** |
| **hsa-miR-novel-chr12_8080** | **5.1000** | **hsa-miR-935** | **2.5455** |
| **hsa-miR-novel-chr16_20127** | **4.6471** | **hsa-miR-22-3p** | **2.4585** |
| **hsa-miR-203a-3p** | **4.2183** | **hsa-miR-novel-chr12_7983** | **2.4000** |
| **hsa-miR-novel-chr6_60431** | **4.1920** | **hsa-miR-450a-5p** | **2.3333** |
| **hsa-miR-139-5p** | **4.0000** | **hsa-miR-378a-3p** | **2.3027** |
| **hsa-miR-novel-chr7_63516** | **3.7000** | **hsa-miR-125a-5p** | **2.2884** |

**Table S2 50 miRNAs were down-regulated in SW620 compared to SW480**

**Annotation：Total RNA from SW480 and SW620 were extracted and used for microRNA sequencing analysis. The top**

**50 miRNAs of 64 were listed in the table.**
