# Supplementary material for: Regulation of cancer stem cell properties, angiogenesis, and vasculogenic mimicry by miR-450a-5p/SOX2 axis in colorectal cancer
Source: Cell Death Dis. 2020 Mar 6;11(3):173. doi: 10.1038/s41419-020-2361-z (PMC7060320; doi:10.1038/s41419-020-2361-z)
Supplement: Supplementary file 4 — Table S3 [file 41419_2020_2361_MOESM4_ESM.docx]

**Table S3 Correlation between the clinicopathologic characteristics and**

**expression of miR-450a-5p**

| **Variables** | | **miR-450a-5p expression** | | ***P-value*** |
| --- | --- | --- | --- | --- |
|  |  | **Score<**3  **Low(n=48)** | **Score**≥3  **High(n=30)** |  |
| **Age(years)** |  |  |  |  |
| < 65 |  | 19 | 9 | 0.391 |
| ≥ 65 |  | 29 | 21 |  |
| **Gender** |  |  |  |  |
| Male |  | 25 | 13 | 0.452 |
| Female |  | 23 | 17 |  |
| **Pathological grade** |  |  |  |  |
| Well-Moderate |  | 39 | 27 | 0.353 |
| Poor |  | 9 | 3 |  |
| **Lymph metastasis** |  |  |  |  |
| no |  | 24 | 23 | **0.019** |
| yes |  | 24 | 7 |  |
| **Distant metastasis** |  |  |  |  |
| no |  | 48 | 29 | 0.385 |
| yes |  | 0 | 1 |  |
| **AJCC stage** |  |  |  |  |
| I/II |  | 24 | 23 | **0.019** |
| III/IV |  | 24 | 7 |  |
| **Tumer size** |  |  |  |  |
| < 5 cm |  | 13 | 15 | **0.040** |
| ≥ 5 cm |  | 35 | 15 |  |
| **Tumor number** |  |  |  |  |
| Single |  | 47 | 29 | 0.999 |
| Multiple |  | 1 | 1 |  |
|  |  |  |  |  |
