# Supplementary figures and images for: Regulation of cancer stem cell properties, angiogenesis, and vasculogenic mimicry by miR-450a-5p/SOX2 axis in colorectal cancer
Source: Cell Death Dis. 2020 Mar 6;11(3):173. doi: 10.1038/s41419-020-2361-z (PMC7060320; doi:10.1038/s41419-020-2361-z)

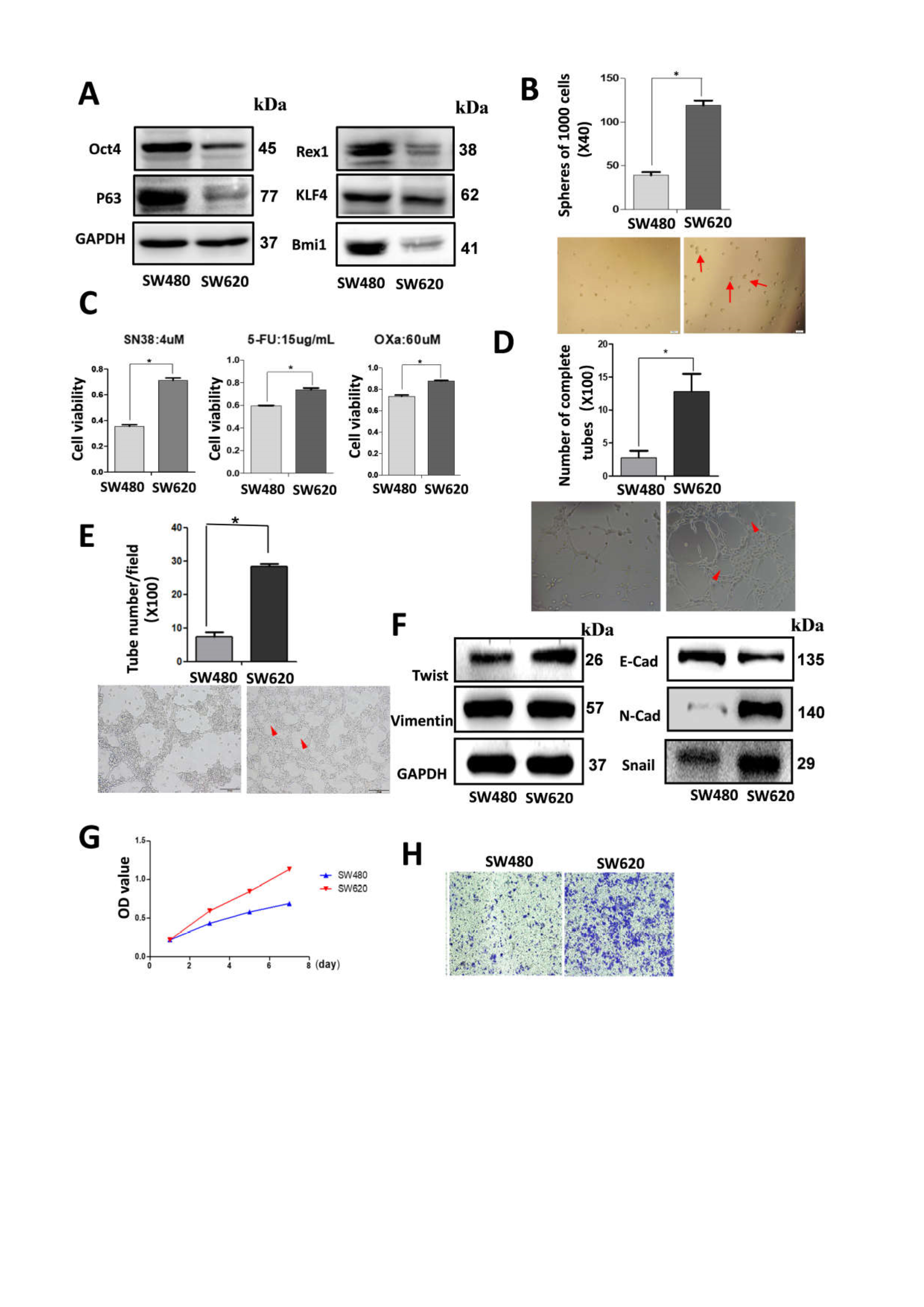

Supplement: Supplementary file 5 — figureS1 [file 41419_2020_2361_MOESM5_ESM.tif]

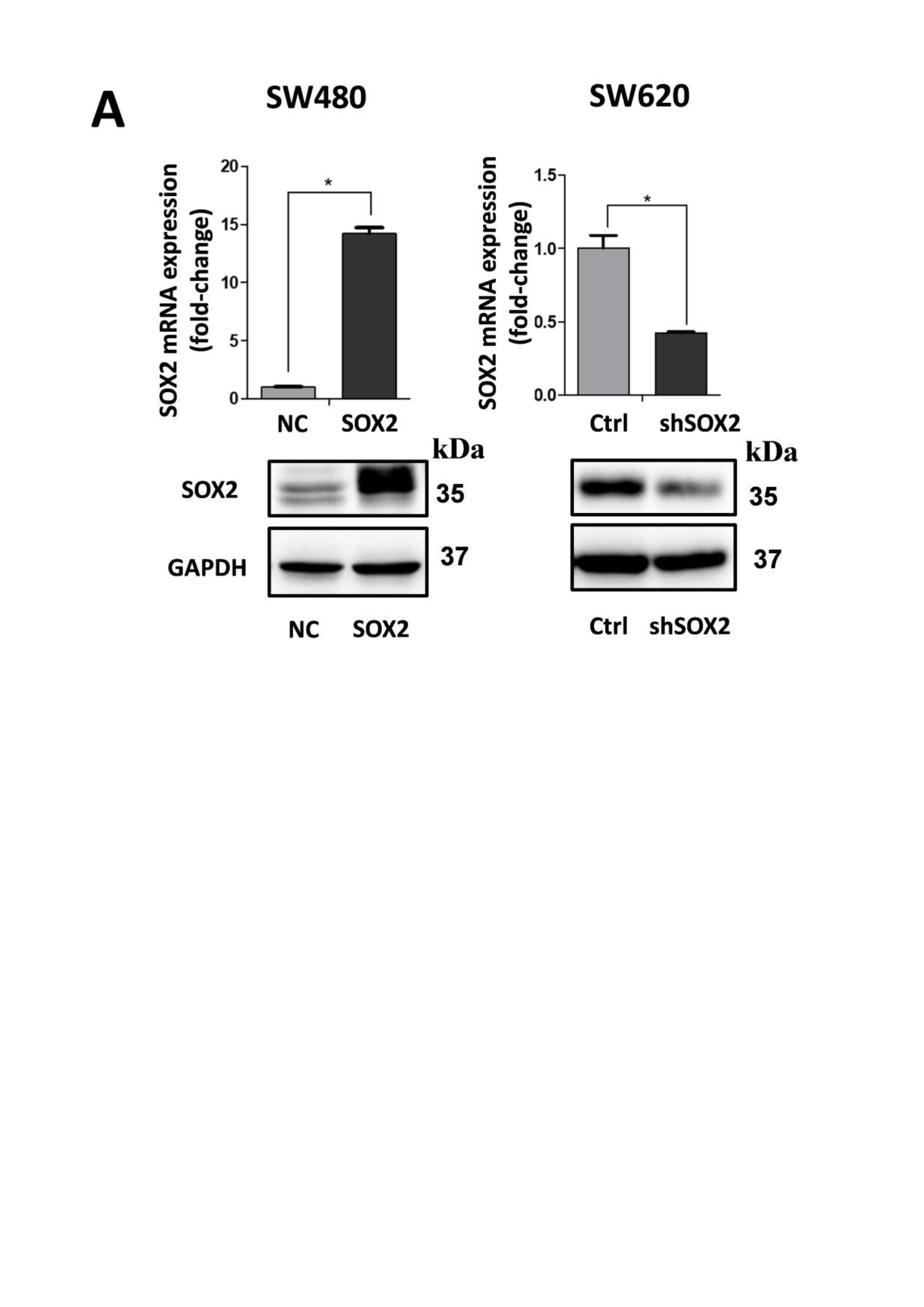

Supplement: Supplementary file 6 — figureS2 [file 41419_2020_2361_MOESM6_ESM.tif]

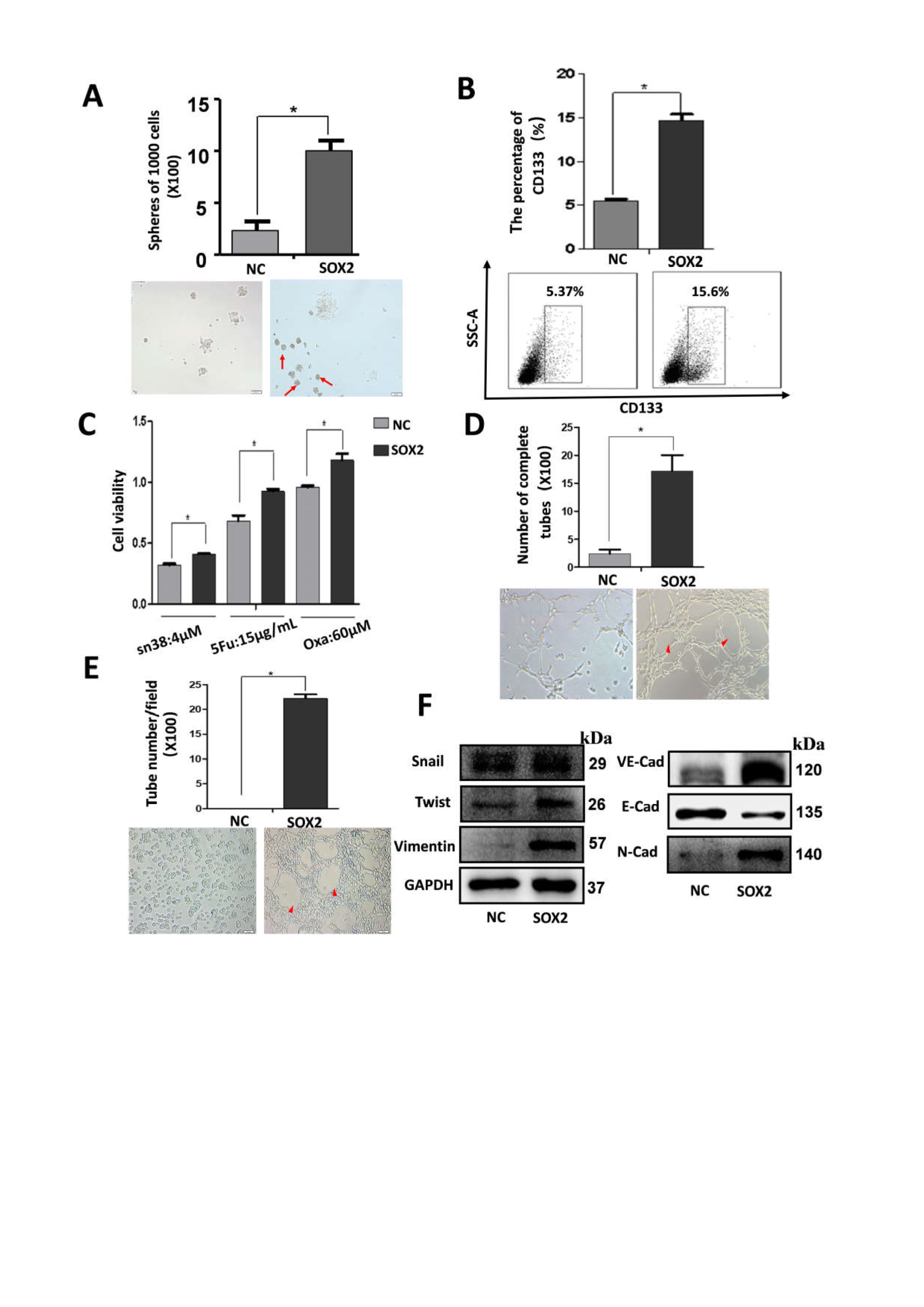

Supplement: Supplementary file 7 — figureS3 [file 41419_2020_2361_MOESM7_ESM.tif]

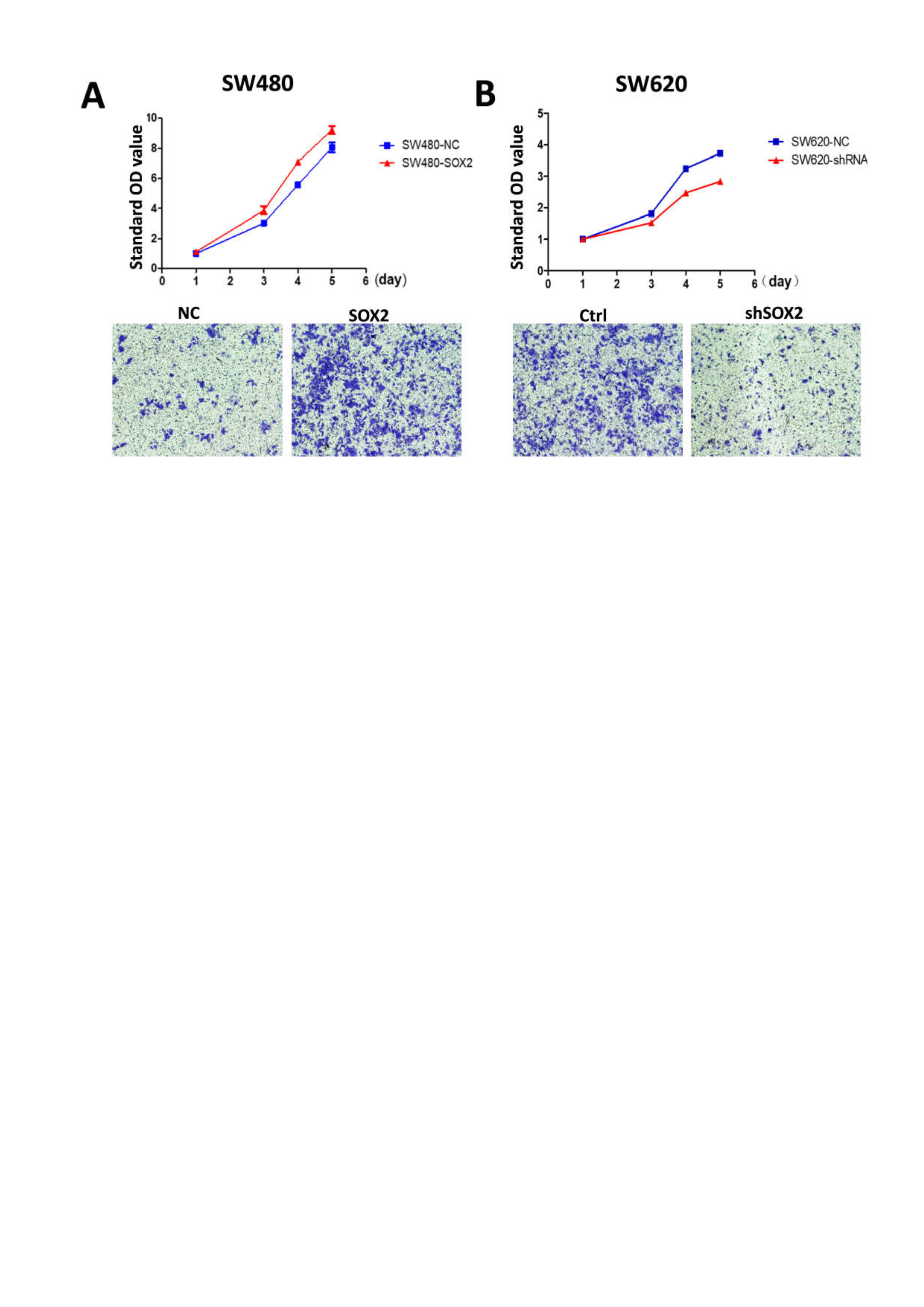

Supplement: Supplementary file 8 — figureS4 [file 41419_2020_2361_MOESM8_ESM.tif]

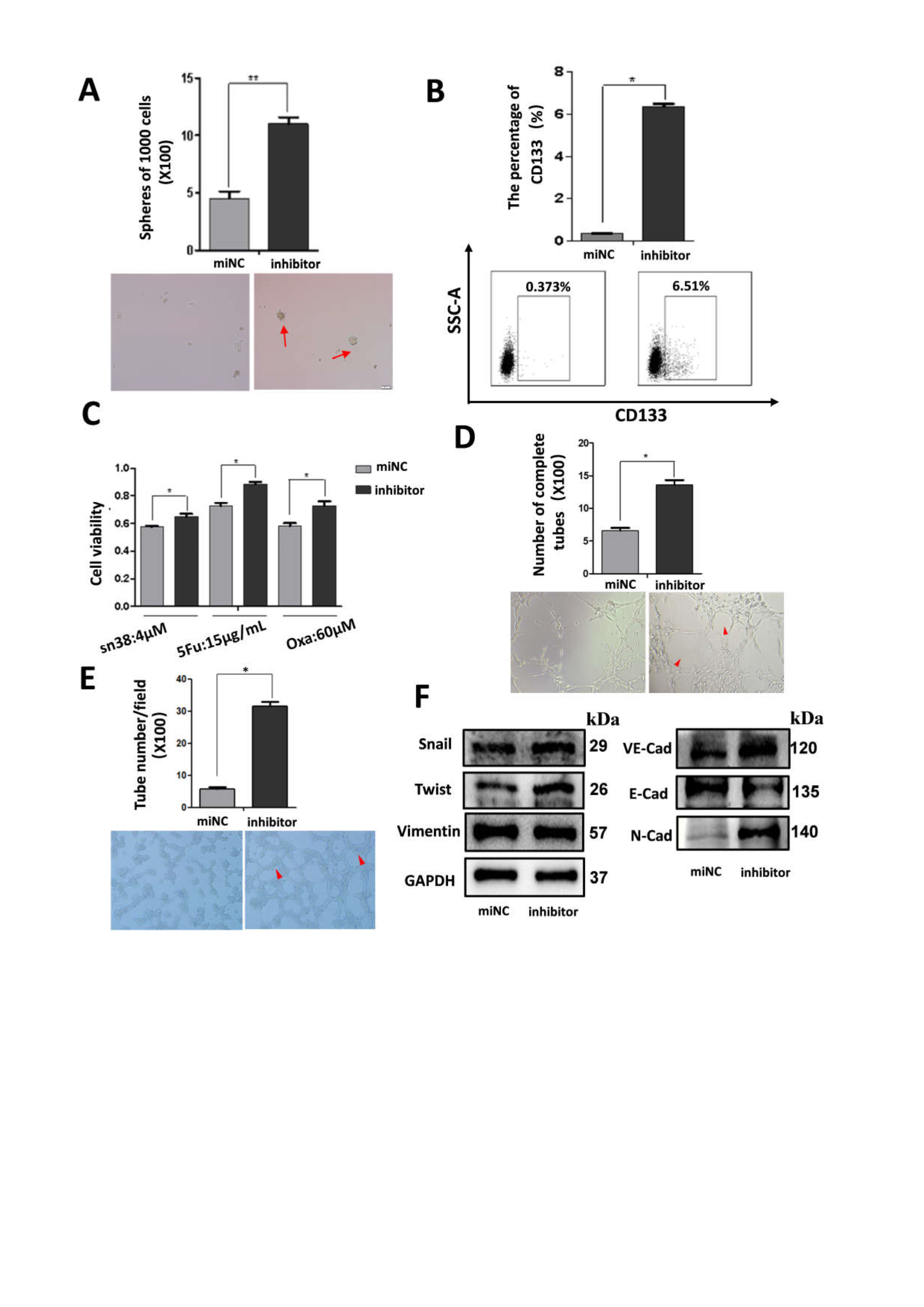

Supplement: Supplementary file 9 — figureS5 [file 41419_2020_2361_MOESM9_ESM.tif]

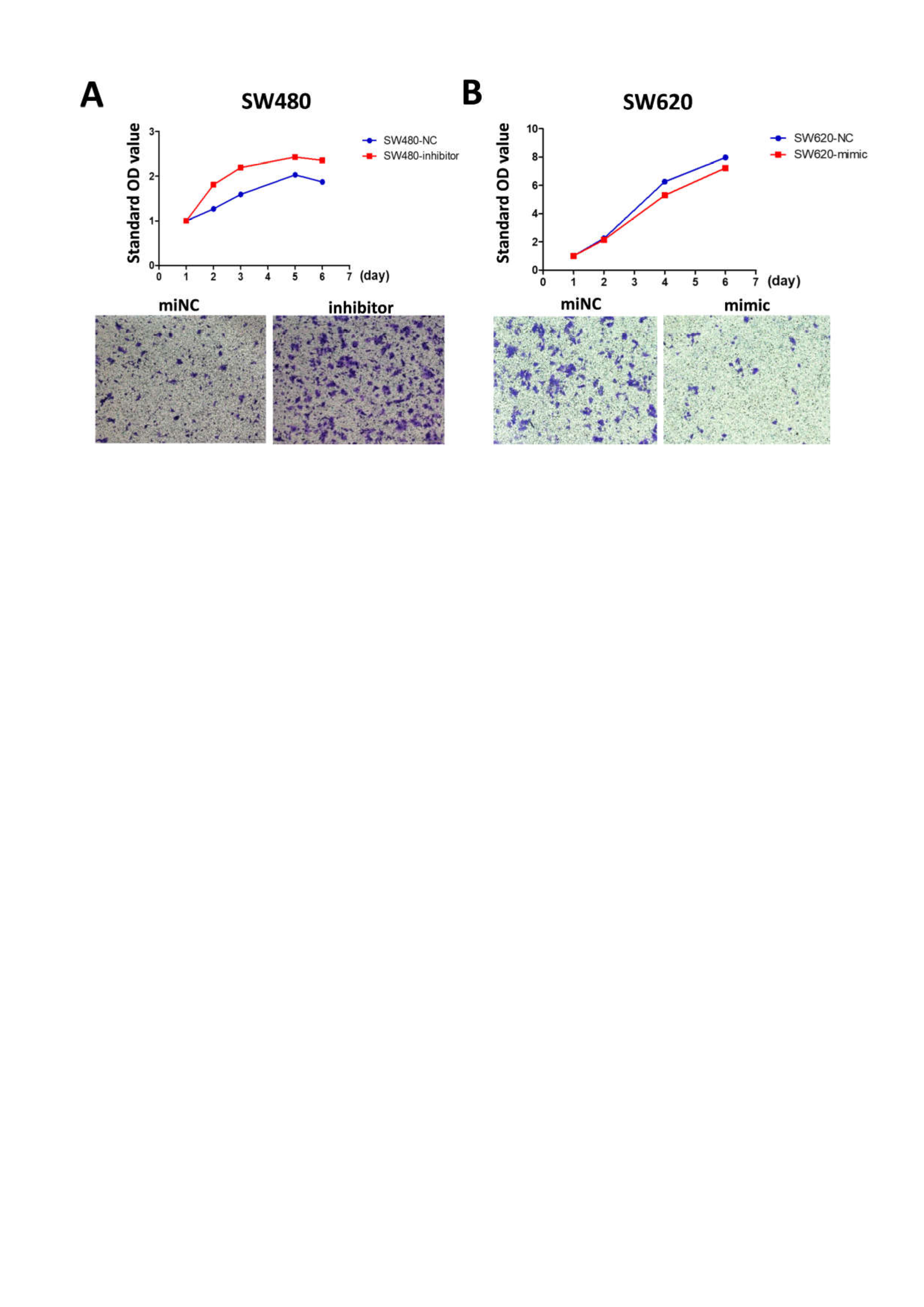

Supplement: Supplementary file 10 — figureS6 [file 41419_2020_2361_MOESM10_ESM.tif]

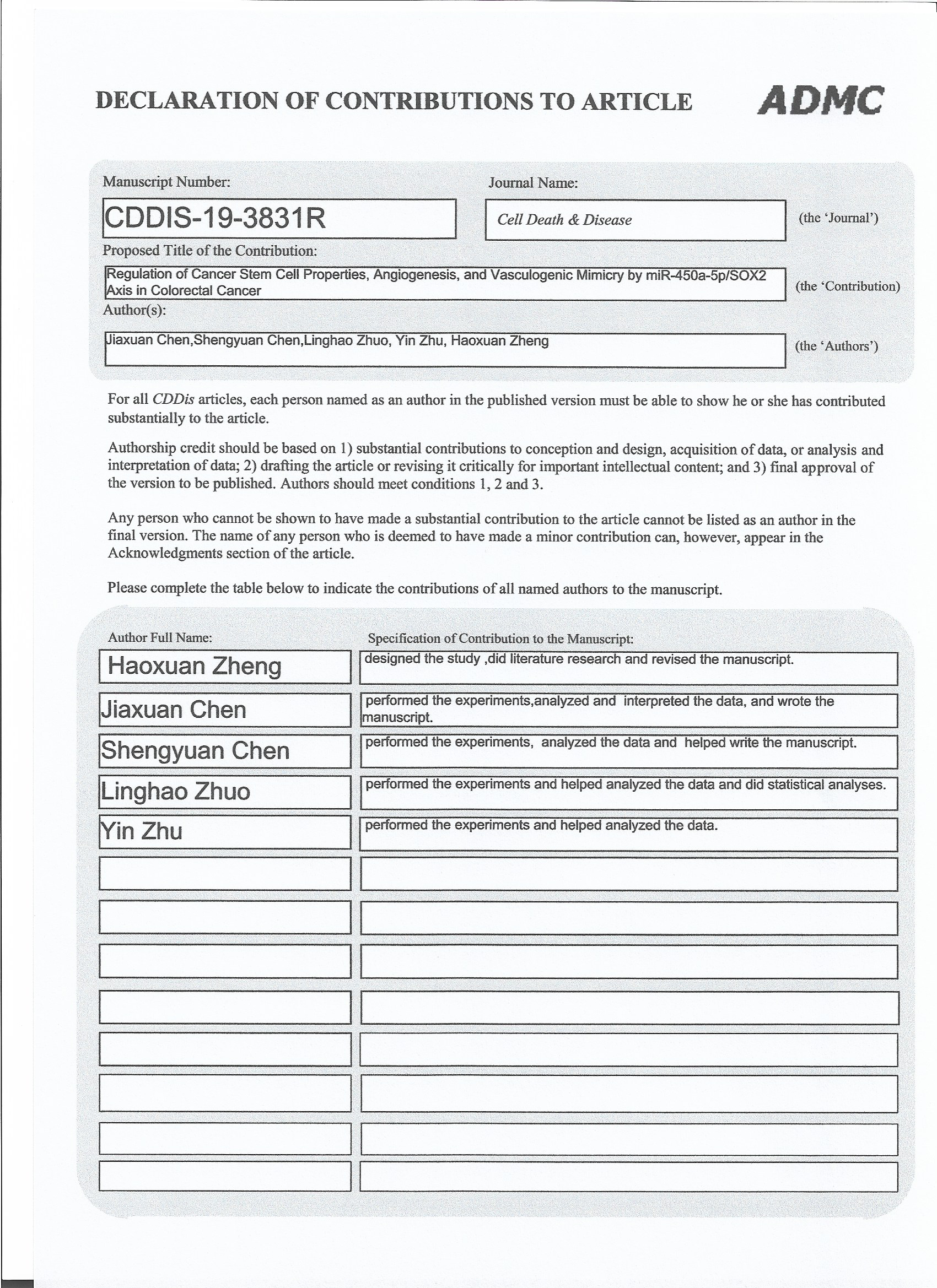

Supplement: Supplementary file 12 — author contribution form-page1 [file 41419_2020_2361_MOESM12_ESM.tif]

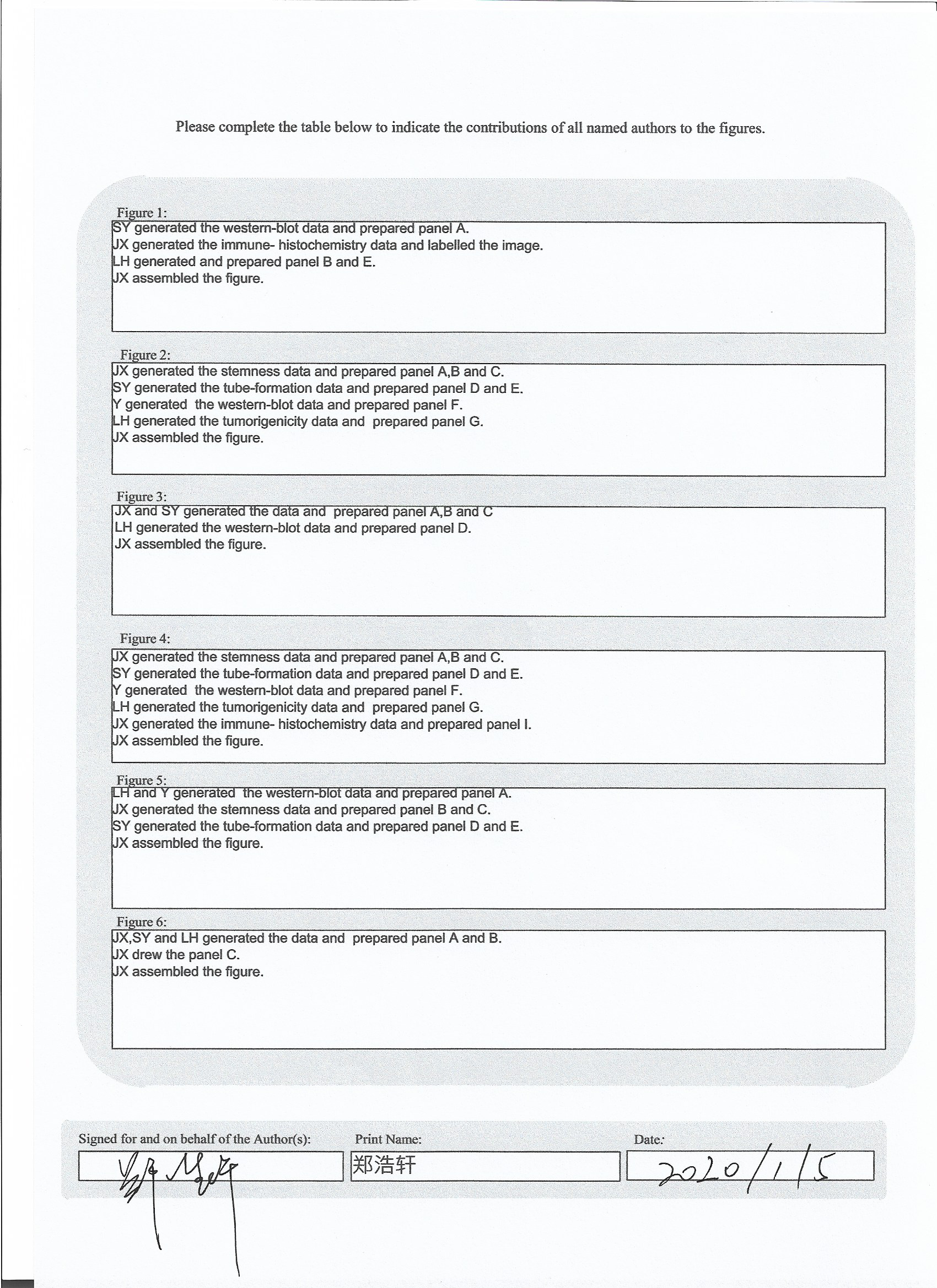

Supplement: Supplementary file 13 — author contribution form-page2 [file 41419_2020_2361_MOESM13_ESM.tif]
